# *DEP1* is involved in regulating the carbon–nitrogen metabolic balance to affect grain yield and quality in rice (*Oriza sativa* L.)

**DOI:** 10.1371/journal.pone.0213504

**Published:** 2019-03-11

**Authors:** Mingzhu Zhao, Minghui Zhao, Shuang Gu, Jian Sun, Zuobin Ma, Lili Wang, Wenjing Zheng, Zhengjin Xu

**Affiliations:** 1 Rice Research Institute, Liaoning Academy of Agricultural Sciences, Shenyang, China; 2 Rice Research Institute, Shenyang Agricultural University, Shenyang, China; China National Rice Research Institute, CHINA

## Abstract

The *DEP1* (*dense and erect panicle 1*) gene, which corresponds to the erect panicle architecture, shows a pleiotropic effect in increasing grain yield and nitrogen use efficiency (NUE) in rice. Nevertheless, it remains unclear whether the carbon−nitrogen metabolic balance changes as the *dep1* allele enhances nitrogen uptake and assimilation. In this study, we generated transgenic Akitakomati plants by overexpressing *dep1* and analyzed the carbon−nitrogen metabolic status, gene expression profiles, and grain yield and quality. Under either low or high nitrogen growth conditions, the carbon−nitrogen metabolic balance of *dep1*-overexpressed lines was broken in stem sheaths and leaves but not in grains; the *dep1*-overexpressed plants showed higher expressions of *glutamine synthetase* (*GS*) and *glutamate synthase* (*GOGAT*) genes than the wildtype, along with increased total nitrogen and soluble protein content in the straw at maturity. However, the *ribulose-1*,*5-bisphosphate carboxylase/oxygenase* (*RUBISCO*) and *phosphoenolpyruvate carboxylase* (*PEPC*) genes were downregulated in *dep1*-overexpressed plants, leading to a decreased carbohydrate content and carbon/nitrogen ratio. Although the unbalanced carbon−nitrogen metabolism decreased the grain-filling rate, grain setting percentage, 1000 grain weight, and grain quality in *dep1*-overexpressed lines, it led to increased grain numbers per panicle and consequently increased grain yield. Our results suggest that an unbalanced carbon−nitrogen metabolic status is a major limiting factor for further improving grain yield and quality in erect panicle varieties.

## Introduction

Nitrogen is an essential nutrient in the growth and development of plants [1]. A great deal of nitrogen fertilizer is applied to fields to maximize grain yield for its significant effect on crop productivity [2]. However, excessive nitrogen fertilizer application results in severe environmental pollution, particularly in aquatic ecosystems [3]. Thus, it is important to optimize the use of nitrogen fertilizers to make agriculture more sustainable. One method of optimization is to increase nitrogen use efficiency (NUE) through genetic improvement, particularly in rice (*Oryza sativa* L.), which would increase grain yield with less nitrogen fertilizer [4].

Inorganic nitrogen is mainly absorbed by ammonium transporters (AMTs) in rice roots and is assimilated via glutamine synthetase (GS) within the plant; its product (glutamine) is digested into glutamate by the GS/GOGAT cycle or into asparagine by asparagine synthetase (As) [5]. In the past few decades, GS was the main focus in crop research due to its key role in controlling nitrogen assimilation [6]. Many previous studies have attempted to overexpress the *GS* gene in rice to obtain transgenic plants with higher NUE; however, *GS*-overexpressed plants exhibit poor plant growth and less grain yield [7–9]. Unbalanced carbon−nitrogen metabolism is the crucial reason for decreased grain yield in *GS*-overexpressed plants [8]. Metabolite profiles of a knockout mutant of rice *GS1;1* have revealed an imbalance in the levels of sugars, amino acids, and metabolites in the tricarboxylic acid cycle [10]. For these reasons, although the activity of GS is related to NUE, it is still difficult to increase rice yield by overexpressing it.

The synthesis of nitrogen-containing compounds, including various amino acids, proteins, or enzymes, requires the incorporation of ammonium into their carbon skeletons. The required energy and carbon skeletons for ammonium assimilation are provided from sucrose, glucose, organic acids, and other carbohydrates [11]. Maintaining an appropriate balance of carbon−nitrogen metabolites plays an important role in plant growth and development [12]. Many studies have indicated that numerous central metabolites involved in carbon and nitrogen metabolism can be altered in parallel rather than antagonistically if the nitrogen or carbon supply is changed [13–15]. In order to determine the carbon and nitrogen metabolism status of plant tissues, the carbon/nitrogen ratio is usually used as an empirical indicator [7–9].

Recently, a major quantitative trait locus for NUE in rice was cloned that is synonymous with the *DENSE AND ERECT PANICLES 1* (*DEP1*) gene [16]; the *DEP1* protein belongs to a γ subunit of the heterotrimeric G protein [17, 18], which not only plays an essential role in nitrogen signaling [16] but is also involved in carbon metabolism [19–21]. Rice plants carrying the gain-of-function *dep1* allele exhibit erect panicles, higher GS activity, and higher nitrogen uptake even under lower nitrogen growth conditions, and consequently, have increased NUE and grain yield [16]. Therefore, a feasible way to increase the grain yield is by introducing the *dep1* allele into varieties with curved panicles. However, it remains unknown whether the carbon−nitrogen metabolic balance and grain quality will change, while NUE and grain yield are improved in transgenic *dep1* plants.

Akitakomati, a *japonica* variety with good grain quality, carries the *DEP1* allele and exhibits curved panicles. A previous study found that the grain yield of Akitakomati is lower than that of some erect panicle varieties with the *dep1* allele [22]. In this study, we introduced *dep1* into Akitakomati by a binary vector, and then analyzed the carbon−nitrogen metabolic status, gene expression profiles, and grain yield and quality of the transgenic plants under low and high nitrogen growth conditions. The aim was to detect the effect of the *dep1* allele on the carbon−nitrogen metabolic balance, yield traits, and grain quality in curve panicle varieties, which may provide a theoretical basis for the improvement of rice varieties.

## Materials and methods

### Plant transformation

**Fig 1:**
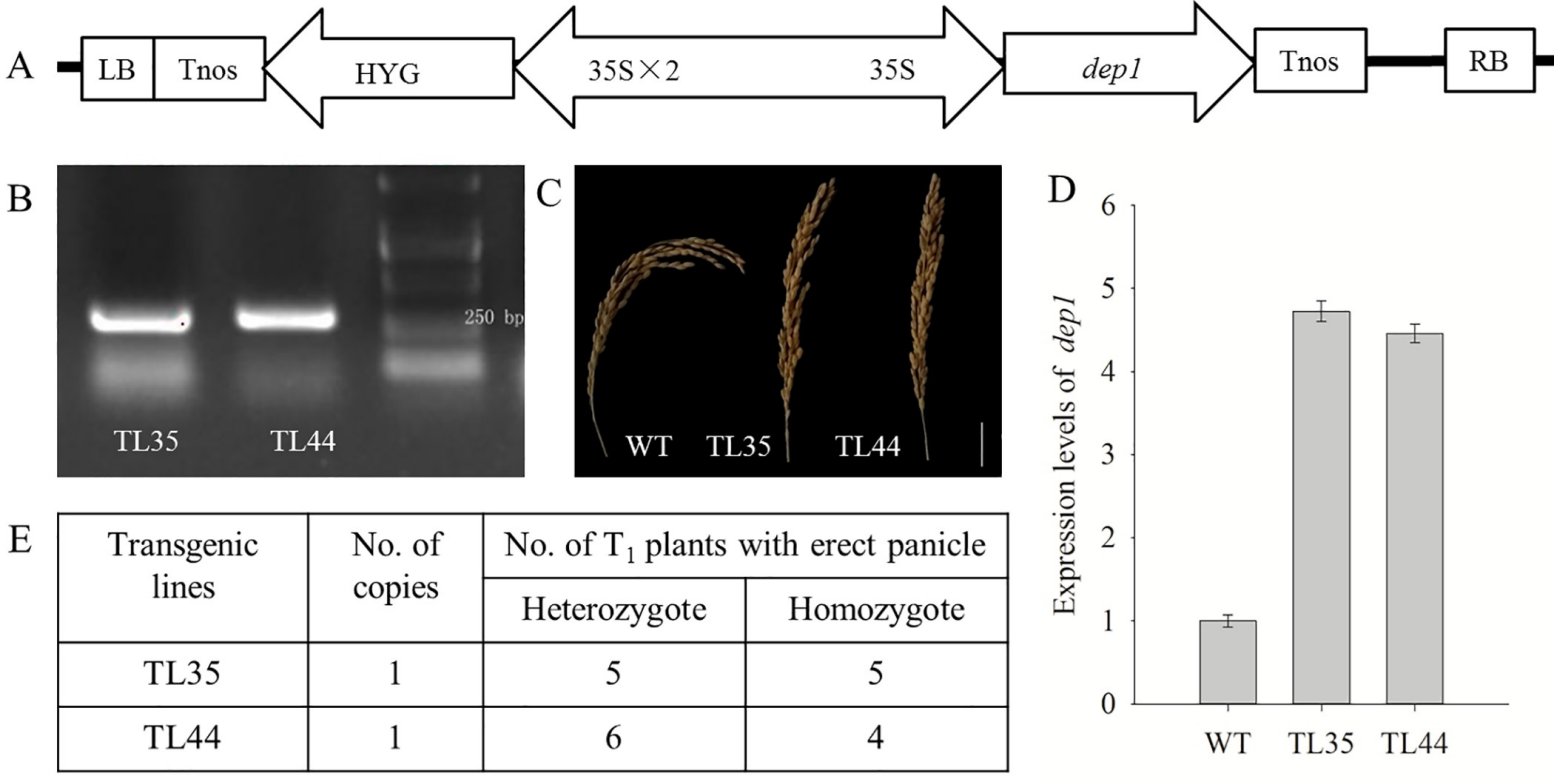
Generation of *dep1* overexpressed plants. (A) The construct of the plasmid containing the CaMV 35S promoter (35S), *dep1* and the terminator (Tnos) between the right (RB) and the left (LB) borders of the T-DNA. The hygromycin resistance gene (*HYG*) was located between the LB and the 35S promoter. (B) Identification of positive transgenic plants in T_0_ generation by PCR. (C) Panicle architecture for wildtype and transgenic T_0_ plants. (D) Expression levels of *dep1* in transgenic T_0_ plants. Expression levels relative to wildtype plants set to be one. Data shown as means ± SD (n = 3). (E) Identification of copy numbers in transgenic T_0_ plants and homozygote in transgenic T_1_ plants by quantitative real-time PCR (S2 and S3 Tables).

Akitakomati carries the *DEP1* allele and exhibits curved panicles. In contrast, Liaojing5 with the *dep1* allele exhibits erect panicles. *DEP1* and *dep1* are a pair of alleles, and the erect panicle is dominant. In this variety, a 12-bp nucleotide sequence replaces a 637-bp region in the middle of exon 5 at the *DEP1* locus. [22–24]. The full coding sequence of *dep1* (FJ039905) was isolated from the cDNA of the erect panicle variety Liaojing 5 (S1 Table) and was ligated into the binary vector pBWA(V)HS that uses the CaMV 35S promoter (Fig 1A). As the *dep1* allele at the *DEP1* locus shows a gain-of-function mutation [24], the binary vector carrying the *dep1* allele was transformed into the curved panicle variety Akitakomati to obtain *dep1*-overexpressed plants (erect panicle mutants) via the *Agrobacterium tumefaciens*-mediated transformation method by Wuhan Biorun Biotechnology Company (www.biorun.net). Twenty-one transgenic plants were obtained from one independent transformation by using the vector pBWA(V)HS. The hygromycin resistance gene (*HYG*) was used as a selectable marker to identify positive transgenic plants by PCR (S1 Table), and copy numbers (S2 Table) were determined by qRT-PCR in the T_0_ generation [25]. Among the 21 transgenic plants, two positive transgenic plants with a single copy, TL35 and TL44, exhibiting erect panicle architecture and high expression of *dep1* (Fig 1B–1D) were used to generate the T_1_ generation. Homozygous T_1_ generation plants (Fig 1E; S3 Table) were identified by qRT-PCR [26], and the seeds were selected and used to generate the T_2_ generation for subsequent study.

### Plant growth conditions

The experiments were conducted in an isolated paddy field at the experimental farm of Shenyang Agricultural University, Shenyang (41.8°N, 123.4°E), China, in the summer of 2016. Seeds of *dep1*-overexpressed plants (TL35 and TL44) in the T_2_ generation and wildtype (Akitakomati) were sown in a seedling nursery on April 26, 2016 with one seedling being transplanted per hill on May 24, 2016. Seedlings were transplanted at 30 cm × 13 cm spacing. The soil contained organic matter of 26.41 g·kg^-1^, total nitrogen of 0.92 g·kg^-1^, available nitrogen of 0.06 g·kg^-1^, available phosphorus of 0.03 g·kg^-1^, available potassium of 0.12 g·kg^-1^, and pH of 5.87. Two nitrogen fertilizer treatments were used, including low nitrogen (LN, 0 kg·ha^-1^ nitrogen, 60 kg·ha^-1^ phosphorus, 100 kg·ha^-1^ potassium) and high nitrogen (HN, 200 kg·ha^-1^ nitrogen, 60 kg·ha^-1^ phosphorus, 100 kg·ha^-1^ potassium) conditions. These fertilizers were applied as a basal dressing using slow-releasing urea, P_2_O_5_, and KCl. The field trials were performed in randomized complete blocks with three replications per line (TL35, TL44, and wildtype), and each plot area was 12 m^2^. Each replication was separated into two nitrogen treatments, and three lines were randomly arranged into each nitrogen treatment.

### Gene expression

The *dep1* allele was expressed in the root, leaf, culm, inflorescence meristem, and young inflorescence, and exhibited the highest expression in the inflorescence meristem at the stage of primary and secondary rachis branch formation [24]. We analyzed the effect of overexpressing the *dep1* allele on the expressions of several genes involved carbon−nitrogen metabolism in leaves at the booting stage when the primary and secondary rachis branches were newly formed. The leaf materials of transgenic lines (TL35, TL44) and wildtype were sampled from three biological replications under the LN and HN conditions, frozen immediately in liquid nitrogen, and stored at –80°C until use. Total RNA was extracted with TriZol reagent (Invitrogen, Germany) according to the manufacturer’s instructions. First strand cDNAs were synthesized from DNaseI-treated total RNA using a Primer Script RT reagent Kit with gDNA Eraser (Takara, Japan) following the manufacturer’s instructions. qRT-PCR was performed in an optical 96-well plate with a real-time PCR system (BIO-RAD). Each reaction contained 3.0 μl of first-strand cDNAs, 2 μl of 200 μM gene-specific primers, and 12.5 μl of 2×SYBR Green Master Mix reagent (Applied Biosystems) in a final volume of 25 μl. Amplification conditions were at 95°C for 3 min, followed by 45 cycles of 95°C for 30 s, 60°C for 30 s, and 72°C for 40 s. The specific primers of tested genes and the reference gene (*ACTIN1*) are listed in S1 Table. The qRT-PCR analysis was performed for each cDNA sample with four replications. Relative expression levels were calculated by 2^-ΔΔCT^ [27]. Normalized expression for TL35 or TL44 was calculated as ΔΔC_T_ = (C_T, *Target*_— C_T, *actin*_) _TL_ — (C_T, *Target*_— C_T, *actin*_) _wildtype_. The results presented are the mean values of three biological replicates for each genotype.

### Chlorophyll content and gas exchange parameters

As differences can usually be observed in phenotype after a change in gene expression levels, we measured the chlorophyll content and gas exchange parameters at the heading stage. One in every 30 plants of TL35, TL44, and wildtype grown under LN and HN conditions were selected to measure the chlorophyll content of flag leaves by a SPAD-502 plus leaf chlorophyll meter (Minolta Camera Co., Osaka, Japan) once every five days after the initial heading stage. At the full heading stage, one in every 12 plants of TL35, TL44, and wildtype grown under LN and HN conditions were selected to measure gas exchange parameters of flag leaves using a LI-6400 portable photosynthesis system (LI-COR, USA). The light intensity was set at 1,500 μmol m^-2^s^-1^. The leaf temperature was kept at 25–30°C, along with a relative humidity of 60%–65%, a CO_2_ concentration of 380 μmol (CO_2_) mol^–1^, and an air flow of 500 μmol s^–1^. All measurements were performed in the morning (9:00–11:30 am).

### Grain-filling rate

Three plants each from TL35, TL44, and the wildtype grown under LN and HN conditions were selected to measure the dry weight of the superior (the top first and last two grains of the upper three primary branches, and the top first grain of the second branch in the upper three primary branches) and inferior grains (the top third and fourth grains of the bottom three primary branches, and the last two grains of the second branch in the bottom three primary branches) every five days after the heading stage. Approximately 100 grains per plant were selected for measurement. Richards’s growth equations as described by Yang et al. [28] were used to simulate the grain-filling process and calculate the grain-filling rate: W = A/ (1+Be^-k*t*^) ^1/N^, where W is the grain weight (g 100 grains^-1^), A is the final grain weight, *t* is the days after heading, and B, *k*, and N are the parameters determined by regression analysis. Grain-filling duration was taken when W was from 10% (*t*_1_) to 90% (*t*_2_) of A. The mean grain-filling rate during the active filling period was calculated from *t*_1_ to *t*_2_.

### Grain yield and quality

At the maturity stage, the above-ground portions of 60 plants of TL35, TL44, and wildtype were harvested from each plot. After counting panicle numbers and measuring plant height, ten average-sized panicles were taken from each plot to observe the panicle length and the numbers of primary and secondary branches. Then, the panicles were hand-threshed and placed in water. Filled grains, sunk in water, were separated from the unfilled grains. To determine dry weight, the filled and unfilled grains were then oven-dried at 80°C for two days. The number of grains per panicle and grain setting percentage were calculated using the above data. The stem sheaths, leaves, and remaining grains of plants were used to determine the biomass, actual yield, and harvest index.

Ten fully filled seeds from each plot were used to measure the grain length (GL), grain width (GW), and grain thickness (GT) using a Vernier caliper. Rough rice for each plot was de-husked and milled to measure the milling quality using a miller according to the National Standards GB/T17891-1999. One hundred milled head rice grains were used to measure the chalkiness grain rate and chalk size. The viscosity of the cooked rice grain was determined using a Rapid Visco Analyzer (RVA-4, Newport Scientific, Sydney, Australia) to obtain profile characteristics according to Standard Method AACC61-02 as recommended by the American Association of Cereal Chemists.

### Carbon and nitrogen metabolites

At the maturity stage, the stem sheaths, leaves, and grains of TL35, TL44, and wildtype grown under LN and HN conditions were used to measure the carbon and nitrogen metabolites. Three samples from stem sheath, leaf, and grain materials from three biological replications were oven-dried at 85°C for 48 h and ground. Samples of ~0.6 g DW (dry weight) were used to measure the total carbon and nitrogen content by a C/N analyzer (Elementar, Vario MAX CN, Germany) according to the manufacturer’s instructions, with L-glutamic acid as a standard. The NUE was calculated with the grain yield divided by the nitrogen accumulation in whole plants.

Dry samples of stem sheaths, leaves, and grain materials from three biological replications were used to measure soluble protein content [29]. Samples of ~0.5 g DW were homogenized by an extraction buffer [10 mM Trizma (pH 7.5), 10 mM MgSO_4_, 5 mM sodium glutamate, 1 mM dithiothreitol, 0.05% (v/v) Triton X-100, and 10% (v/v) glycerol]. Then the homogenates were centrifuged at 2,000 rpm at 4°C for 30 min. The supernatant was used to measure the soluble protein content by Coomassie Brilliant Blue G-250 reagent (Sigma) with four replications for each sample.

Dry samples of stem sheaths, leaves, and grain materials from three biological replications were used to measure soluble carbohydrate content. Samples of ~0.2 g DW were homogenized by 6 ml of 80% ethanol at 80°C for 30 min. The homogenates were centrifuged at 2,000 rpm at 4°C for 30 min, and the supernatant was collected three times, filtered by activated carbon, and diluted in 80% ethanol to 50 ml. The extract was used to measure the sucrose content by the resorcinol-photometric method and the soluble sugar content by the anthrone-photometric method [30]. In addition, the oven-dried residue was homogenized by 2 ml distilled water at 100°C for 20 min, and 2 ml of 9.2 mM perchloric acid was added. The homogenates were centrifuged in 2,000 rpm at 4°C for 30 min, and the supernatant was collected in duplicate. The extract was used to measure the starch content by the anthrone-photometric method [30] with four replications for each sample.

### Statistical analysis

Data analysis was conducted for each trait by analysis of variance (ANOVA) in a general linear model by SPSS 19.0 (SPSS, Inc., Chicago. USA). The treatments with different amounts of nitrogen fertilizer and genotypes were considered as fixed effects. Probability values of less than 0.05 were considered to be significant. Means from three replicates were subjected to the Duncan’s multiple range tests at the *P* < 0.05 level.

## Results

### The *dep1*-overexpressed plants exhibited erect panicles

Using the *A*. *tumefaciens*-mediated method, the pBWA(V)HS vector carrying the *dep1* allele and 35S promoter (Fig 1A) was transformed into the curved panicle variety Akitakomati. Two positive transgenic plants, TL35 and TL44, were identified in the T_0_ generation by the selectable marker *HYG* (Fig 1B). Although the transgenic plants TL35 and TL44 contained the wildtype allele (*DEP1*), they exhibited erect panicle architecture (Fig 1C), which was mainly attributed to the higher expression level of the transformed *dep1* allele in leaves, which is a gain-of-function (Fig 1D). Homozygous plants were obtained in the T_1_ generation from the single-copy transgenic plants TL35 and TL44 (Fig 1E) and were used to generate the T_2_ generation.

### Physiological traits in *dep1*-overexpressed lines

**Fig 2:**
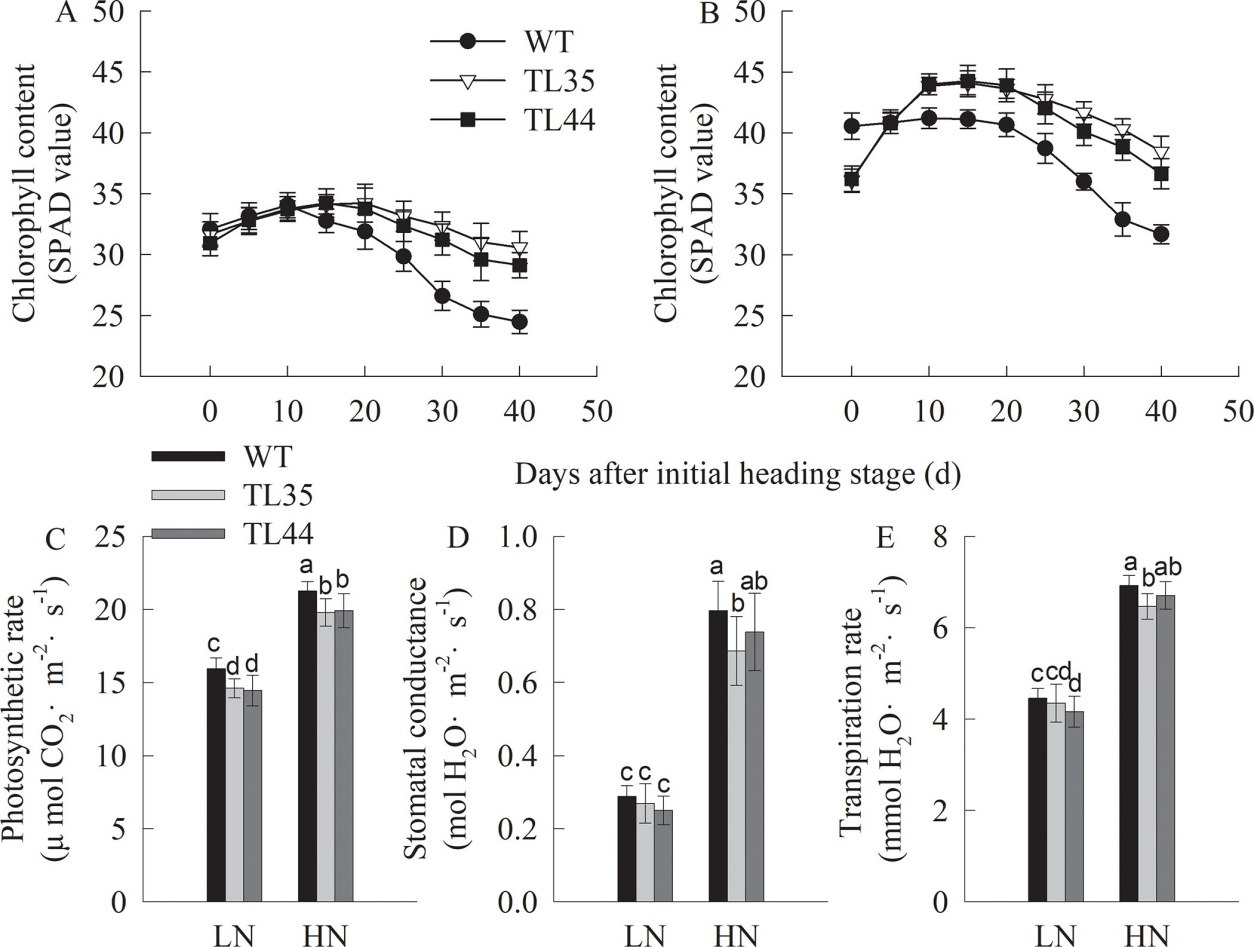
**Chlorophyll content (A and B), photosynthetic rate (C), stomatal conductance (D) and transpiration rate (E) of flag leaves at heading stage in wildtype (WT) and transgenic lines (TL35 and TL44) under low nitrogen (LN) and high nitrogen (HN) conditions.** Data shown as mean ± SD (*n* = 30 or 12 plants). Different letters for the mean values indicate significant differences at *P* < 0.05 by Duncan’s multiple range tests.

The chlorophyll content and gas exchange parameters were compared between transgenic lines and the wildtype after the initial heading stage (Fig 2). The chlorophyll content of flag leaves reached the maximum at 15 and 10 d after the initial heading date under LN (Fig 2A) and HN (Fig 2B) conditions, respectively, and then began to decrease gradually. During this period, the chlorophyll content of transgenic lines (TL35 and TL44) carrying the *dep1* allele declined slowly compared with the wildtype. However, transgenic lines (TL35 and TL44) did not exhibit a significant increase in photosynthetic rate, stomatal conductance, or transpiration rate compared with the wildtype under LN and HN conditions at the full heading stage (Fig 2C–2E).

Compared with the wildtype, transgenic lines (TL35 and TL44) exhibited higher total nitrogen and soluble protein content in stem sheaths and leaves under LN and HN conditions at the maturity stage (Table 1). In contrast, these transgenic lines had lower total carbon, starch, sucrose, and soluble sugar content in stem sheaths and leaves, which led to a decrease in the carbon/nitrogen ratio in these tissues under LN and HN conditions. There was no significant difference in most carbon−nitrogen metabolites in grains between transgenic lines and the wildtype.

**Table 1:** Carbon and nitrogen metabolites at maturity stage in the wildtype (WT) and transgenic lines (TL35 and TL44) under low nitrogen (LN) and high nitrogen (HN) conditions.

Trait	Tissue	LN			HN		
		WT	L35	L44	WT	L35	L44
Total nitrogen content (%)	Stem sheath	0.39e	0.48d	0.49d	0.63c	0.81a	0.69b
	Leaf	1.02e	1.30c	1.15d	1.50b	1.63a	1.66a
	Grain	1.03b	1.07b	1.06b	1.16a	1.18a	1.18a
Total carbon content (%)	Stem sheath	37.15a	36.46b	35.81c	37.40a	36.34b	36.31b
	Leaf	38.09ab	37.36bc	37.52bc	38.89a	37.66bc	36.62c
	Grain	38.22d	38.57d	39.52b	39.04c	39.17bc	40.53a
Carbon/nitrogen ratio	Stem sheath	94.84a	76.65b	73.29b	59.78c	44.80e	52.62d
	Leaf	37.54a	28.65c	32.70b	26.00d	23.07e	22.08e
	Grain	37.18a	36.09ab	37.34a	33.55c	33.22c	34.43bc
Soluble protein content (mg ·g^-1^ DW)	Stem sheath	15.93d	18.02bc	17.87c	17.40bc	19.07a	18.85ab
	Leaf	13.45bc	12.80c	13.77ab	13.20bc	14.00a	13.33ab
	Grain	13.19a	13.11a	13.42a	12.55a	13.24a	14.01a
Starch content (mg ·g^-1^ DW)	Stem sheath	212.66a	180.30b	171.07b	145.67c	111.01d	106.88d
	Leaf	125.79a	103.71bc	98.51cd	113.28b	98.25cd	88.94d
	Grain	272.13b	274.77b	273.43b	300.41a	307.60a	304.21a
Sucrose content (mg ·g^-1^ DW)	Stem sheath	94.01b	62.98cd	57.97d	133.64a	86.62b	73.13c
	Leaf	56.08a	31.81c	26.82c	41.71b	28.74c	26.82c
	Grain	44.33bc	50.45b	57.29a	38.86cd	36.08d	46.24b
Soluble sugar content (mg ·g^-1^ DW)	Stem sheath	190.77b	137.68c	132.71c	242.74a	118.87d	104.47e
	Leaf	90.67a	46.68c	35.18d	70.03b	30.58e	22.70f
	Grain	57.08b	60.14a	64.34b	45.20c	47.13c	47.19c

Data are shown as the means estimated from three plots (each plot contained 20 randomly mixed plant materials) per line per fertilizer. Different letters following the mean values indicate significant differences at *P* < 0.05 by Duncan’s multiple range tests.

Nitrogen absorption and carbohydrate assimilation of these lines were also analyzed at the maturity stage (S4 Table). Compared with the wildtype, transgenic lines (TL35 and TL44) had higher nitrogen accumulation in whole plants under LN and HN conditions, whereas most of the carbohydrate accumulation of these transgenic lines was decreased in stem sheaths and leaves but increased in grains.

### Gene expression patterns in *dep1*-overexpressed lines

**Fig 3:**
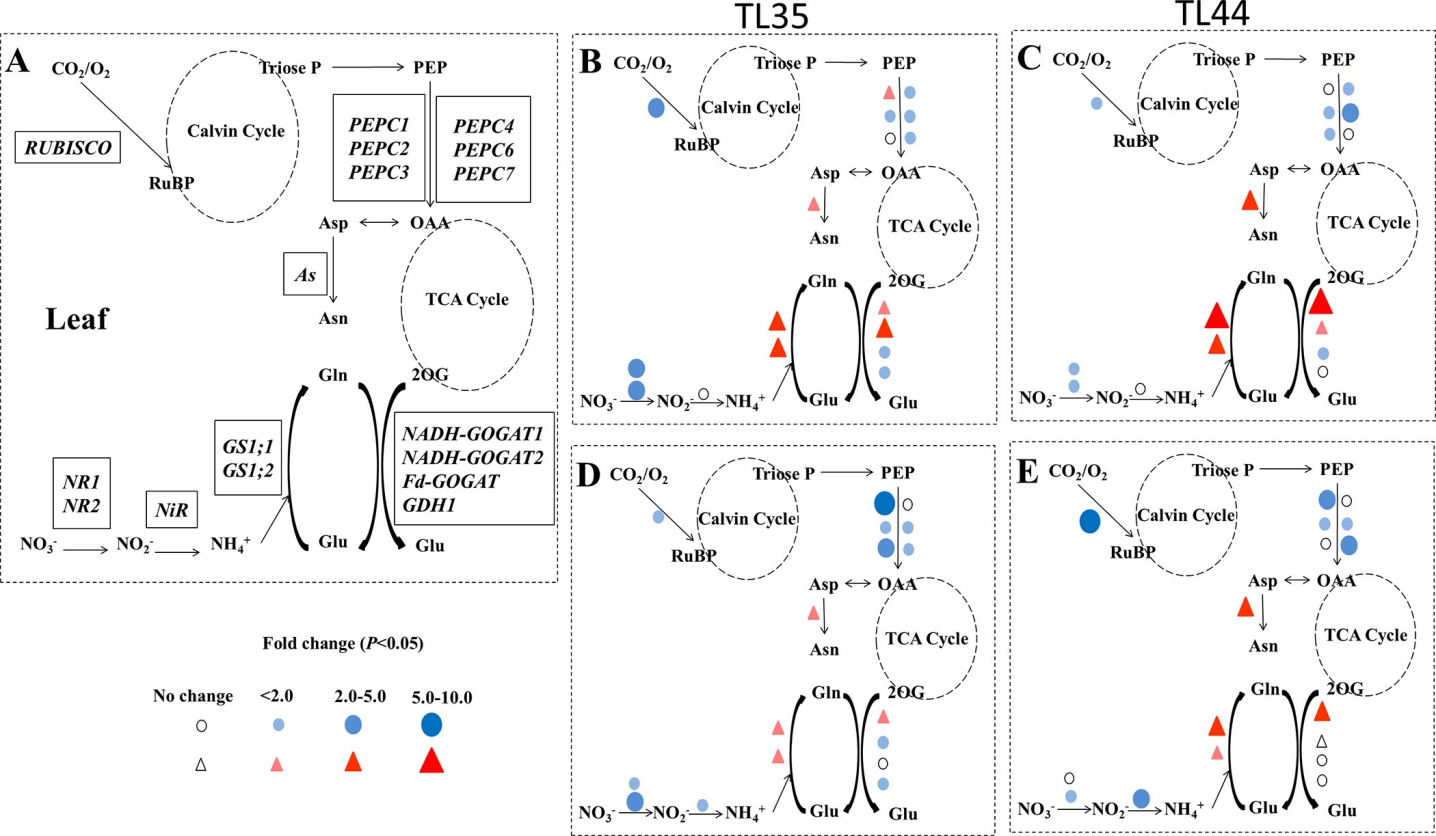
Fold change corresponding to the ratio of the gene expression level in transgenic lines (TL35 and TL44) relative to the wildtype plants. (A) Diagrammatic representation of key genes involved in the carbon and nitrogen metabolic pathway in rice plants. *NR*, nitrate reductase; *NiR*, nitrite reductase; *GS*, glutamine synthetase; *GOGAT*, glutamate synthase; *GDH*, glutamate dehydrogenase; *AS*, asparagine synthetase; *RUBISCO*, ribulose-1,5-bisphosphate carboxylase/oxygenase; *PEPC*, phosphoenolpyruvate carboxylase. Prominent changes in the gene expression levels in TL35 plants compared to wildtype plants at the booting stage under low nitrogen (B) and high nitrogen (C) conditions, and in TL44 plants compared to wildtype plants under low nitrogen (D) and high nitrogen (E) conditions. Red and blue dots indicate up- and down-regulated genes, respectively. The gene expression level was measured from three biological replications and each sample was measured at least five times.

Key genes in the carbon−nitrogen metabolic pathway were detected at the booting stage; these genes had different expression patterns between transgenic lines TL35 and TL44 and the wildtype plants (Fig 3 and S1 Fig). Under LN conditions, compared with the wildtype, the expression levels of *GS1;1*, *GS1;2*, *NADH-GOGAT1*, *NADH-GOGAT2*, *AS*, and *PEPC1* were significantly increased in the transgenic lines TL35 and TL44, whereas the expressions of *NR1*, *NR2*, *Fd-GOGAT*, *GDH1 RUBISCO*, *PEPC2*, *PEPC3*, *PEPC4*, *PEPC6*, and *PEPC7* were significantly decreased (Fig 3B). Under HN conditions, compared with the wildtype, the expression levels of *GS1;1*, *GS1;2*, *NADH-GOGAT1*, and *AS* were significantly increased in the transgenic lines TL35 and TL44, whereas those of *NR1*, *NR2*, *NiR*, *NADH-GOGAT2*, *GDH1*, *RUBISCO*, *PEPC1*, *PEPC2*, *PEPC3*, *PEPC6*, and *PEPC7* were significantly decreased (Fig 3C).

### Grain yield and quality in transgenic *dep1* lines

**Fig 4:**
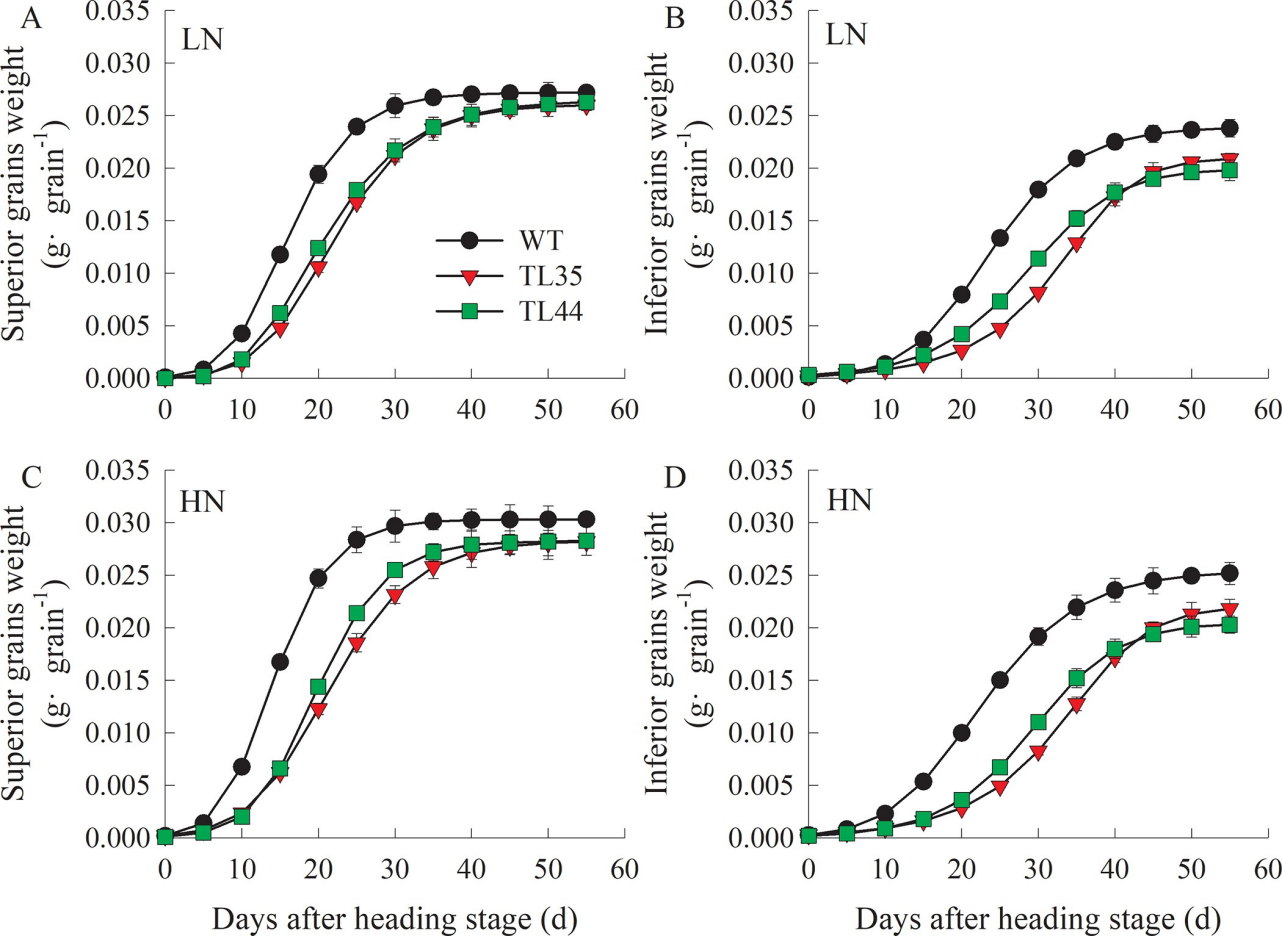
**Accumulations of grain weight after heading stage in the wildtype (WT) and transgenic lines (TL35 and TL44) under low nitrogen (A and B) and high nitrogen (C and D) conditions.** Data shown as mean ± SD (*n* = 3 plants).

The *dep1*-overexpressed lines (TL35 and TL44) exhibited erect panicle types along with a shorter plant height and panicle length, and had increased primary and secondary panicle branches and grain density (Table 2). Under LN conditions, transgenic lines (TL35 and TL44) showed increased grain yields of 27.07% and 34.53% compared to the wildtype, which was mainly attributed to the higher number of panicles per plant, the number of grains per panicle, and biomass. Under HN conditions, the grain yields of transgenic lines (TL35 and TL44) were 14.82% and 13.08% higher than that of the wildtype due to the raised grain numbers per panicle and harvest index. These transgenic lines also exhibited higher NUE than the wildtype under LN and HN conditions. However, the grain setting percentage and 1000 grain weight were significantly decreased in transgenic lines (TL35 and TL44) under either LN or HN conditions. There was a difference in grain weight accumulation after the heading stage between transgenic line (TL35 or TL44) and the wildtype (Fig 4). Grain-filling dynamic analysis for transgenic lines TL35 and TL44 showed smaller initial filling power (R_0_), maximum grain-filling rate (GR_max_), mean grain-filling rate (GR_mean_), and longer time reaching the maximum filling rate (T_max_) and active filling period (D) for both superior and inferior grains, compared with those of wildtype under both LN and HN conditions (S5 Table).

**Table 2:** Agronomic traits at maturity stage in the wildtype (WT) and transgenic lines (TL35 and TL44) under low nitrogen (LN) and high nitrogen (HN) conditions.

Trait	LN			HN		
	WT	TL35	TL44	WT	TL35	TL44
Plant height (cm)	85.40c	77.02d	77.02d	108.86a	91.48b	91.48b
No. of panicles per plant	6.86c	9.23b	9.51b	13.50a	13.79a	13.87a
Panicle length (cm)	17.15b	15.05d	15.06d	18.04a	16.37c	16.70bc
No. of primary panicle branches	8.00c	10.00b	10.00b	10.00b	11.00a	11.00a
No. of secondary panicle branches	10.50d	13.47c	13.57c	14.57b	19.4a	20.17a
Grain density (g·cm^-1^)	4.21d	6.01b	6.04b	5.34c	7.11a	7.16a
Number of grains per panicle	72.13d	90.33c	90.73c	96.10b	116.23a	119.5a
Grain setting percentage (%)	96.84a	92.81b	93.22b	92.36b	88.21c	87.75c
1000-grains weight (g)	26.02ab	24.87c	24.66c	26.49a	25.38bc	25.22bc
Yield (t·ha^-1^)	3.62d	4.60c	4.87c	7.49b	8.60a	8.47a
Biomass (t·ha^-1^)	6.29c	7.54b	7.84b	13.60a	13.67a	13.68a
Harvest index	0.58ab	0.61a	0.62a	0.55b	0.63a	0.62a
NUE	61.69b	70.19a	70.65a	57.76b	58.35b	61.38b

Data are shown as the means estimated from three plots (each plot comprised 60 plants) per line per fertilizer. Different letters following the mean values indicate significant differences at *P* < 0.05 by Duncan’s multiple range tests.

We further determined the grain appearance, milling, and cooking quality in transgenic lines (TL35 and TL44) and the wildtype (Table 3). Under LN and HN conditions, only transgenic line TL44 showed decreased grain length, grain length/width ratio, and head rice rate compared with the wildtype. However, some RVA profile parameters, including the peak viscosity, cool paste viscosity, breakdown value, and consistence value were decreased in transgenic lines (TL35 and TL44), whereas the setback viscosity and peak time were increased compared with wildtype, particularly under HN conditions.

**Table 3:** Grain quality traits at maturity stage in the wildtype (WT) and transgenic lines (TL35 and TL44) under low nitrogen (LN) and high nitrogen (HN) conditions.

Trait	LN			HN		
	WT	TL35	TL44	WT	TL35	TL44
Grain length (mm)	7.06ab	7.10ab	6.89bc	7.22a	6.98ab	6.67c
Grain width (mm)	3.10c	3.24ab	3.21bc	3.15bc	3.35a	3.18bc
Grain thickness (mm)	2.14c	2.22b	2.13c	2.14c	2.28a	2.17bc
Length/width ratio	2.28ab	2.20abc	2.16bc	2.30a	2.09c	2.12c
Chalky grain percentage (%)	15.17cd	13.68d	13.43d	22.89a	21.27ab	18.50bc
Chalkiness degree	3.13d	3.63cd	3.13d	6.13a	5.64ab	4.83bc
Brown rice rate (%)	77.44a	78.50a	77.45a	77.56a	78.11a	78.56a
White rice rate (%)	66.33a	68.83a	67.65a	69.11a	66.56a	69.33a
Head rice rate (%)	57.73c	62.33ab	61.83ab	60.47bc	61.71b	64.67a
Peak viscosity (cP)	3588a	3009bc	3080b	3387a	3108b	2812c
Hot paste viscosity (cP)	2167ab	2322ab	2421ab	2142b	2431a	2331a
Cool paste viscosity (cP)	4533a	3996bc	4064bc	4298ab	4100b	3746c
Breakdown value (cP)	1420a	687b	659b	1245a	676b	481b
Setback viscosity (cP)	946b	986a	984a	911c	992a	934b
Consistence value (cP)	2366a	1673b	1643b	2156a	1668b	1415b
Pasting temperature (°C)	70.00ab	68.30b	67.97b	71.28a	69.95ab	69.00b
Peak time (min)	6.38b	6.53ab	6.73ab	6.42b	6.62ab	6.93a

Data are shown as the means estimated from three plots (each plot contained 60 plants) per line per fertilizer. Different letters following the mean values indicate significant differences at *P* < 0.05 by Duncan’s multiple range tests.

## Discussion

Rice plants carrying the dominant *DEP1* allele (*dep1*) have higher expression levels of *GS1;1* and GS activity, exhibiting nitrogen-insensitive vegetative growth coupled with increased nitrogen uptake and assimilation [16]. However, many studies have found that the balance of carbon−nitrogen metabolism can be broken if the nitrogen metabolism activity is increased in *GS*-overexpressed plants [8–10]. In this study, the carbon/nitrogen ratio of *dep1*-overexpressed lines was lower in stem sheaths and leaves than that of the wildtype under either LN or HN conditions, which was not only attributed to the increased total nitrogen content but also decreased total carbon content. Similar results in previous studies have also shown that more carbohydrate or nitrogen accumulating in plants automatically results in lower concentrations of other components [31–34]. However, the carbon/nitrogen ratio is sometimes considered to be a poor indicator of the carbon and nitrogen metabolism status of plant tissues [34]. Thus, to provide evidence of unbalanced carbon−nitrogen metabolism in *dep1*-overexpressed lines, we showed an increase in soluble protein content and decreases in starch, sucrose, and soluble sugar content in the straw of these lines. The gene expression patterns involved in carbon-nitrogen metabolism were further analyzed. The *dep1*-overexpressed lines had higher expressions of *GS* and *GOGAT* genes, and thus showed higher nitrogen metabolic activity than the wildtype under either LN or HN conditions. Meanwhile, the genes involved in carbon metabolism, such as *RUBISCO* and *PEPC*, were suppressed, which caused unbalanced carbon−nitrogen metabolism in stem sheaths and leaves under both LN and HN conditions.

Previous studies have shown that unbalanced carbon−nitrogen metabolic status can result in some negative effects, such as poor plant growth, inferior photosynthetic capacity, lower nitrogen transfer ability, and decreased yield [8–10, 35, 36], but the *dep1* allele can lead to an increased number of grains per panicle, and consequently, increase grain yield [24, 37]. Moreover, the *dep1* allele makes panicles dense and erect, which causes improvements in the population structure, light-interception capacity, and ecological environment [38, 39]. In this study, overexpressed *dep1* led to greater nitrogen absorption and NUE, and the *dep1*-overexpressed lines (TL35 and TL44) exhibited higher numbers of panicles per plant, grain numbers per panicle, biomass, and grain yield under LN conditions, and had higher grain numbers per panicle, harvest index, and grain yield under HN conditions. Meanwhile, despite the unbalanced carbon−nitrogen metabolism in stem sheaths and leaves, the *dep1*-overexpressed lines also had higher carbon accumulation in grains and whole plants, leading to higher grain yield than the wildtype under LN and HN conditions.

Some previous studies have reported that the *dep1* allele can decrease the grain-filling percentage and the 1000 grains weight [23, 37, 40]. *DEP1*, like *GS3* that encodes a γ subunit of the heterotrimeric G protein, regulates grain size and shape by protein−protein interactions [41, 42]. Overexpression of the *DEP1* allele leads to large grains, whereas that of the *dep1* allele results in small grains [41]. *DEP1* interacts with *RGB1* to promote grain growth, while *GS3* regulates grain size by repressing the function of *DEP1* [41]. This study showed that sink capacity improved by increasing the grain number per panicle in the *dep1*-overexpressed lines under LN or HN conditions, but the source was insufficient due to a poor photosynthetic rate, resulting in a decrease of the grain-filling rate, grain-setting percentage, grain length, and 1000 grains weight. Moreover, there was a decrease in peak viscosity, cool paste viscosity, breakdown value, and consistence value, and an increase in setback viscosity and peak time in grains of *dep1*-overexpressed lines under LN or HN conditions. The viscidity of cooked rice is negatively correlated with setback viscosity and breakdown value [43], and the amylose content of cooked rice is positively correlated with setback viscosity [44]. Rice varieties with good eating quality (such as Akitakomati used in this study) usually have higher peak viscosity and breakdown values, but smaller setback viscosity compared to common varieties [45]. This study suggests that unbalanced carbon−nitrogen metabolism resulted in decreased grain quality in *dep1*-overexpressed lines.

In conclusion, metabolic and gene expression profile analysis showed that the carbon−nitrogen metabolic status was unbalanced in stem sheaths and leaves but not in grains of *dep1*-overexpressed lines. This status did not result in decreased grain yield, though it reduced the grain-filling rate, grain setting percentage, 1000 grain weight, and grain quality. These results can explain the reasons for poor grain quality in most of erect panicle varieties. They also suggest that some other genes related to grain size or grain quality should be aggregated with the *dep1* allele to improve grain yield and grain quality together for super rice breeding.

## Supporting information

S1 Table Primer sequences used in this study. (DOCX)

S2 Table Copy number of exogenous gene in transgenic T_0_ plants identified by quantitative real-time PCR. (DOCX)

S3 Table Zygosity analysis of exogenous gene in transgenic T_1_ plants identified by quantitative real-time PCR. (DOCX)

S4 Table Carbon and nitrogen accumulation at maturity stage in the wildtype (WT) and transgenic lines (TL35 and TL44) under low nitrogen (LN) and high nitrogen (HN) conditions. (DOCX)

S5 Table Parameters of grain filling in the wildtype (WT) and transgenic lines (TL35 and TL44) under low nitrogen (LN) and high nitrogen (HN) conditions. (DOCX)

S1 Fig **Fold change corresponding to the ratio of the gene expression level in transgenic lines TL35 (A) and TL44 (B) relative to the wildtype plants**.
(TIF)
