# Supplementary material for: DEP1 is involved in regulating the carbon–nitrogen metabolic balance to affect grain yield and quality in rice (Oriza sativa L.)
Source: PLoS One. 2019 Mar 11;14(3):e0213504. doi: 10.1371/journal.pone.0213504 (PMC6411142; doi:10.1371/journal.pone.0213504)
Supplement: S1 Table — (DOCX) [file pone.0213504.s001.docx]

**S1 Table.** Primer sequences used in this study.

| Gene | Forward primer (5'-3') | Reverse primer (5'-3') |  |
| --- | --- | --- | --- |
| *dep1* | ATGGGGGAGGAGGCGGTGGTGATG | CCTAGATGTTGAAGCAGGTGCAG | Clone |
| *HYG* | ACGGTGTCGTCCATCACAGTTTGCC | TTCCGGAAGTGCTTGACATTGGGGA | Identify |
| *SPS* | TTGCGCCTGAACGGATAT | CGGTTGATCTTTTCGGGATG | Identify(reference gene) |
| *dep1* | GCGAGATCACGTTCCTCAAG | TGCAGTTTGGCTTACAGCAT | Expression analysis |
| *NR1* | CGCTGCTGCCGCTCATCCG | CCGTGCCCATGGCCAGAC | Expression analysis |
| *NR2* | GAGTTCTTCAACGGGGAGAT | CGAGCAGGAAGAAGAACTTG | Expression analysis |
| *NiR* | CGAGGAGTAGGAACACAG | TGTCGTCTACTTTACAAGGA | Expression analysis |
| *GS1;1* | CACCAACAAGAGGCACAATG | ACTCCCACTGTCCTGGCAT | Expression analysis |
| *GS1;2* | TGTTTCTCCTCATCCCTGC | TCACAGTCCTCGCTTTGC | Expression analysis |
| *NADH-GOGAT1* | GTGCAGCCTGTTGCAGCATAAA | CGGCATTTCACCATGCAAATC | Expression analysis |
| *NADH-GOGAT2* | CCTGTCGAAGGATGATGAAGGTGAAACC | TGCATGGCCCTACTATCTTCGCATCA | Expression analysis |
| *Fd-GOGAT* | GCATACTTGTGAAGCACCGAAGTG | CTGCAAATAGCAACCTAGCGTCAG | Expression analysis |
| *GDH1* | CATCTGATCATCTCCCTGTT | TTCAGGCAATTCATCACTAC | Expression analysis |
| *As* | TTACCTAAGCACATTCTATACAG | CCTTCAATCCATCAATCCAA | Expression analysis |
| *RUBISCO* | ACATTCCGTGTTGCTGCAGAG | TGCAACAGTTCAACCGCTAGG | Expression analysis |
| *PEPC1* | ACATTCCGTGTTGCTGCAGAG | TGCAACAGTTCAACCGCTAGG | Expression analysis |
| *PEPC2* | CAGAAGCACGCAAGCATTAGG | CGCGAGAATCTCTCTCTGAAGG | Expression analysis |
| *PEPC3* | ACCGGTCCATTGTCTTCCAAG | CGTTTTGATGGCCTACTTCCAA | Expression analysis |
| *PEPC4* | TGGATGAGATGGCTGTTGTGG | TTCTGTCTCAGGTGTTGCCGA | Expression analysis |
| *PEPC6* | ATGTCTGCCAGGCTTACACGAT | CGGCTTAGACCAGTCCATGATC | Expression analysis |
| *PEPC7* | GAGTATTTCCGCCTTGCAACAC | ACGGAGTGATTCAATGCCTCC | Expression analysis |
| *ACTIN1* | CATCGCCGAGTACTTCTAC | ATCCAAATGTTCCAGAGGCG | Expression analysis(reference gene) |
