# Supplementary material for: DEP1 is involved in regulating the carbon–nitrogen metabolic balance to affect grain yield and quality in rice (Oriza sativa L.)
Source: PLoS One. 2019 Mar 11;14(3):e0213504. doi: 10.1371/journal.pone.0213504 (PMC6411142; doi:10.1371/journal.pone.0213504)
Supplement: S2 Table — (DOCX) [file pone.0213504.s002.docx]

**S2 Table.** Copy number of exogenous gene in transgenic T_0_ plants identified by quantitative real-time PCR.

| Line | C_T_ value | |  | Template amounts (TA) | |  | 2×TA*_HYG_*/TA*_SPS_* | Copy number |
| --- | --- | --- | --- | --- | --- | --- | --- | --- |
|  | *HYG* | *SPS* |  | *HYG* | *SPS* |  |  |  |
| WT | 36.67 | 27.73 |  | 0×C | 280×C |  | 0.00 | 0 |
| TL33 | 24.29 | 28.95 |  | 140×C | 132×C |  | 2.13 | 2 |
| TL34 | 24.93 | 29.65 |  | 91×C | 86×C |  | 2.11 | 2 |
| TL35 | 25.28 | 28.85 |  | 73×C | 144×C |  | 1.03 | 1 |
| TL36 | 23.36 | 28.53 |  | 266×C | 171×C |  | 3.12 | 3 |
| TL37 | 22.26 | 27.74 |  | 565×C | 276×C |  | 4.10 | 4 |
| TL38 | 22.74 | 27.23 |  | 408×C | 378×C |  | 2.16 | 2 |
| TL39 | 24.08 | 27.53 |  | 164×C | 314×C |  | 1.04 | 1 |
| TL40 | 24.11 | 29.31 |  | 160×C | 107×C |  | 3.01 | 3 |
| TL41 | 22.76 | 27.17 |  | 403×C | 392×C |  | 2.06 | 2 |
| TL42 | 22.46 | 26.76 |  | 493×C | 505×C |  | 1.96 | 2 |
| TL43 | 24.60 | 28.26 |  | 114×C | 202×C |  | 1.13 | 1 |
| TL44 | 23.10 | 26.53 |  | 317×C | 576×C |  | 1.10 | 1 |
| TL45 | 24.12 | 27.64 |  | 158×C | 294×C |  | 1.08 | 1 |
| TL46 | 21.45 | 25.81 |  | 985×C | 902×C |  | 2.19 | 2 |

C_T_ value, Cycle thresholds of hygromycin resistance gene (*HYG*) and reference gene (*SPS*). Template amounts were calculated by standard curve. C_T_(*HYG*)=10^[-3.374^^×TA+31.538]^; C_T_(*SPS*)=10^[-3.776×TA +36.954]^. C represented the assumed initial template amounts of exogenous and reference gene of the standard sample. Data shown as mean (*n* = 3).
