# Supplementary material for: DEP1 is involved in regulating the carbon–nitrogen metabolic balance to affect grain yield and quality in rice (Oriza sativa L.)
Source: PLoS One. 2019 Mar 11;14(3):e0213504. doi: 10.1371/journal.pone.0213504 (PMC6411142; doi:10.1371/journal.pone.0213504)
Supplement: S3 Table — (DOCX) [file pone.0213504.s003.docx]

**S3 Table.** Zygosity analysis of exogenous gene in transgenic T_1_ plants identified by quantitative real-time PCR.

| Plant | C_T_ value | | Fold change | Zygosity |
| --- | --- | --- | --- | --- |
|  | *HYG* | *SPS* |  |  |
| TL35(T_0_) | 26.54 | 25.66 | 1.00 | heterozygote |
| TL35-1 | 26.35 | 26.48 | 2.02 | homozygote |
| TL35-2 | 26.72 | 25.94 | 1.07 | heterozygote |
| TL35-3 | 26.24 | 26.32 | 1.94 | homozygote |
| TL35-4 | 26.06 | 26.14 | 1.94 | homozygote |
| TL35-5 | 27.56 | 26.72 | 1.03 | heterozygote |
| TL35-6 | 27.32 | 26.48 | 1.03 | heterozygote |
| TL35-7 | 27.31 | 26.44 | 1.01 | heterozygote |
| TL35-8 | 27.15 | 27.25 | 1.98 | homozygote |
| TL35-9 | 26.11 | 26.21 | 1.98 | homozygote |
| TL35-10 | 27.38 | 26.53 | 1.02 | heterozygote |
| TL44(T_0_) | 26.64 | 27.61 | 1.00 | heterozygote |
| TL44-1 | 26.57 | 27.61 | 1.05 | heterozygote |
| TL44-2 | 25.38 | 27.34 | 1.99 | homozygote |
| TL44-3 | 26.18 | 27.15 | 1.00 | heterozygote |
| TL44-4 | 25.98 | 27.97 | 2.02 | homozygote |
| TL44-5 | 25.87 | 26.83 | 0.99 | heterozygote |
| TL44-6 | 25.46 | 26.43 | 1.00 | heterozygote |
| TL44-7 | 26.39 | 27.37 | 1.01 | heterozygote |
| TL44-8 | 26.18 | 27.19 | 1.03 | heterozygote |
| TL44-9 | 26.11 | 28.11 | 2.04 | homozygote |
| TL44-10 | 25.48 | 27.46 | 2.02 | homozygote |

C_T_ value, cycle thresholds of hygromycin resistance gene (*HYG*) and reference gene (*SPS*). Fold change was calculated by 2^-∆∆CT^, ∆∆C_T_= (∆C_T_ *_HYG_* -∆C_T_ *_SPS_*) _T1_ - (∆C_T_ *_HYG_* -∆C_T_ *_SPS_*) _calibration_. Transgenic T_0_ plants were heterozygote, which were as calibration to calculate the fold change of *HYG* in transgenic T_1_ plants relative to that in transgenic T_0_ plants. Data shown as mean (*n* = 3).
