# Supplementary material for: DEP1 is involved in regulating the carbon–nitrogen metabolic balance to affect grain yield and quality in rice (Oriza sativa L.)
Source: PLoS One. 2019 Mar 11;14(3):e0213504. doi: 10.1371/journal.pone.0213504 (PMC6411142; doi:10.1371/journal.pone.0213504)
Supplement: S4 Table — (DOCX) [file pone.0213504.s004.docx]

**S4 Table.** Carbon and nitrogen accumulation at maturity stage in the wildtype (WT) and transgenic lines (TL35 and TL44) under low nitrogen (LN) and high nitrogen (HN) conditions.

| Trait (plant^-1^) | Tissue | LN | | |  | HN | | |
| --- | --- | --- | --- | --- | --- | --- | --- | --- |
|  |  | WT | TL35 | TL44 |  | WT | TL35 | TL44 |
| Nitrogen accumulation (g) | Stem sheath | 0.03c | 0.04c | 0.05c |  | 0.12a | 0.12a | 0.10b |
|  | Leaf | 0.02c | 0.03b | 0.03bc |  | 0.08a | 0.09a | 0.09a |
|  | Grain | 0.15d | 0.20c | 0.21c |  | 0.35b | 0.41a | 0.40a |
|  | Total | 0.20d | 0.27c | 0.28c |  | 0.55b | 0.61a | 0.60a |
| Carbon accumulation (g) | Stem sheath | 3.27c | 3.37c | 3.37c |  | 7.09a | 5.41b | 5.51b |
|  | Leaf | 0.72b | 0.94b | 0.93b |  | 2.13a | 2.03a | 2.07a |
|  | Grain | 5.53d | 7.09c | 7.70c |  | 11.70b | 13.48a | 13.73a |
|  | Total | 9.52c | 11.41b | 12.00b |  | 20.92a | 20.92a | 21.31a |
| Soluble protein accumulation (g) | Stem sheath | 0.14c | 0.17c | 0.17c |  | 0.33a | 0.28b | 0.29b |
|  | Leaf | 0.03b | 0.03b | 0.03b |  | 0.07a | 0.08a | 0.08a |
|  | Grain | 0.19d | 0.24cd | 0.26c |  | 0.38b | 0.46a | 0.47a |
|  | Total | 0.36c | 0.44b | 0.46b |  | 0.78a | 0.82a | 0.84a |
| Starch accumulation (g) | Stem sheath | 1.87b | 1.67b | 1.61b |  | 2.76a | 1.65b | 1.63b |
|  | Leaf | 0.24c | 0.26c | 0.24c |  | 0.62a | 0.53b | 0.50b |
|  | Grain | 3.94d | 5.05c | 5.32c |  | 9.00b | 10.58a | 10.29a |
|  | Total | 6.05c | 6.98b | 7.18b |  | 12.38a | 12.76a | 12.42a |
| Sucrose accumulation (g) | Stem sheath | 0.83c | 0.58d | 0.55d |  | 2.53a | 1.29b | 1.12b |
|  | Leaf | 0.11c | 0.08d | 0.07d |  | 0.23a | 0.16b | 0.15b |
|  | Grain | 0.64d | 0.93c | 1.12b |  | 1.16b | 1.24b | 1.56a |
|  | Total | 1.57c | 1.59c | 1.73c |  | 3.92a | 2.69b | 2.83b |
| Soluble sugar accumulation (g) | Stem sheath | 1.68b | 1.27c | 1.25c |  | 4.60a | 1.77b | 1.58b |
|  | Leaf | 0.17b | 0.12d | 0.09d |  | 0.38a | 0.17bc | 0.13cd |
|  | Grain | 0.83d | 1.11c | 1.25b |  | 1.36b | 1.62a | 1.60a |
|  | Total | 2.68c | 2.50c | 2.59c |  | 6.34a | 3.56b | 3.31b |

Data are shown as the mean value estimated from three plots (each plot contained 20 randomly mixed plant materials) per line per fertilizer. Different letters indicate statistical difference significance at P < 0.05 by Duncan’s multiple range tests.
