# Supplementary material for: DEP1 is involved in regulating the carbon–nitrogen metabolic balance to affect grain yield and quality in rice (Oriza sativa L.)
Source: PLoS One. 2019 Mar 11;14(3):e0213504. doi: 10.1371/journal.pone.0213504 (PMC6411142; doi:10.1371/journal.pone.0213504)
Supplement: S5 Table — (DOCX) [file pone.0213504.s005.docx]

**S5 Table.** Parameters of grain filling in the wildtype (WT) and transgenic lines (TL35, TL44) under low nitrogen (LN) and high nitrogen (HN) conditions.

| Treatment | Grain type | Line | A  (g·grain^-1^) | B | K | N | R^2^ | R_0_ | T_max_  (d) | GR_max_  (g·grain^-1^·d^-1^) | W_max_  (g·grain^-1^) | GR_mean_  (g·grain^-1^·d^-1^) | D  (d) |
| --- | --- | --- | --- | --- | --- | --- | --- | --- | --- | --- | --- | --- | --- |
| LN | Superior | WT | 0.0272 | 8.32 | 0.203 | 0.398 | 0.997 | 0.510 | 14.97 | 0.0017 | 0.0117 | 0.0012 | 23.63 |
|  |  | TL35 | 0.0261 | 11.62 | 0.162 | 0.416 | 0.997 | 0.389 | 20.55 | 0.0013 | 0.0113 | 0.0009 | 29.83 |
|  |  | TL44 | 0.0264 | 1.30 | 0.137 | 0.106 | 0.997 | 1.289 | 18.30 | 0.0013 | 0.0102 | 0.0009 | 30.75 |
|  | Inferior | WT | 0.0239 | 24.07 | 0.161 | 0.612 | 0.996 | 0.263 | 22.81 | 0.0011 | 0.0110 | 0.0007 | 32.45 |
|  |  | TL35 | 0.0210 | 261.90 | 0.185 | 1.196 | 0.996 | 0.155 | 29.13 | 0.0009 | 0.0109 | 0.0006 | 34.55 |
|  |  | TL44 | 0.0200 | 267.87 | 0.183 | 1.318 | 0.998 | 0.139 | 29.04 | 0.0008 | 0.0106 | 0.0006 | 36.26 |
| HN | Superior | WT | 0.0303 | 10.52 | 0.232 | 0.473 | 0.993 | 0.490 | 13.37 | 0.0021 | 0.0134 | 0.0014 | 21.32 |
|  |  | TL35 | 0.0283 | 14.53 | 0.161 | 0.547 | 0.996 | 0.294 | 20.37 | 0.0013 | 0.0127 | 0.0009 | 31.64 |
|  |  | TL44 | 0.0283 | 29.75 | 0.204 | 0.606 | 0.997 | 0.337 | 19.09 | 0.0016 | 0.0130 | 0.0011 | 25.55 |
|  | Inferior | WT | 0.0254 | 9.34 | 0.137 | 0.504 | 0.994 | 0.272 | 21.31 | 0.0010 | 0.0113 | 0.0007 | 36.55 |
|  |  | TL35 | 0.0221 | 21.33 | 0.131 | 0.549 | 0.994 | 0.239 | 27.94 | 0.0008 | 0.0100 | 0.0006 | 38.92 |
|  |  | TL44 | 0.0205 | 306.57 | 0.186 | 1.224 | 0.995 | 0.152 | 29.70 | 0.0009 | 0.0107 | 0.0006 | 34.67 |

A, growth capacity of a grain; B, initial parameter; K, growth rate parameter; N, shape parameter; R_0_, the initial filling power; T_max_, the time reaching the maximum filling rate; GR_max_, maximum grain-filling rate; W_max_, weight of a grain at the time of maximum grain-filling rate; GR_mean_, mean grain-filling rate; D, active filling period (from 10%-90% of a grain weight).
