# Supplementary figures and images for: DEP1 is involved in regulating the carbon–nitrogen metabolic balance to affect grain yield and quality in rice (Oriza sativa L.)
Source: PLoS One. 2019 Mar 11;14(3):e0213504. doi: 10.1371/journal.pone.0213504 (PMC6411142; doi:10.1371/journal.pone.0213504)

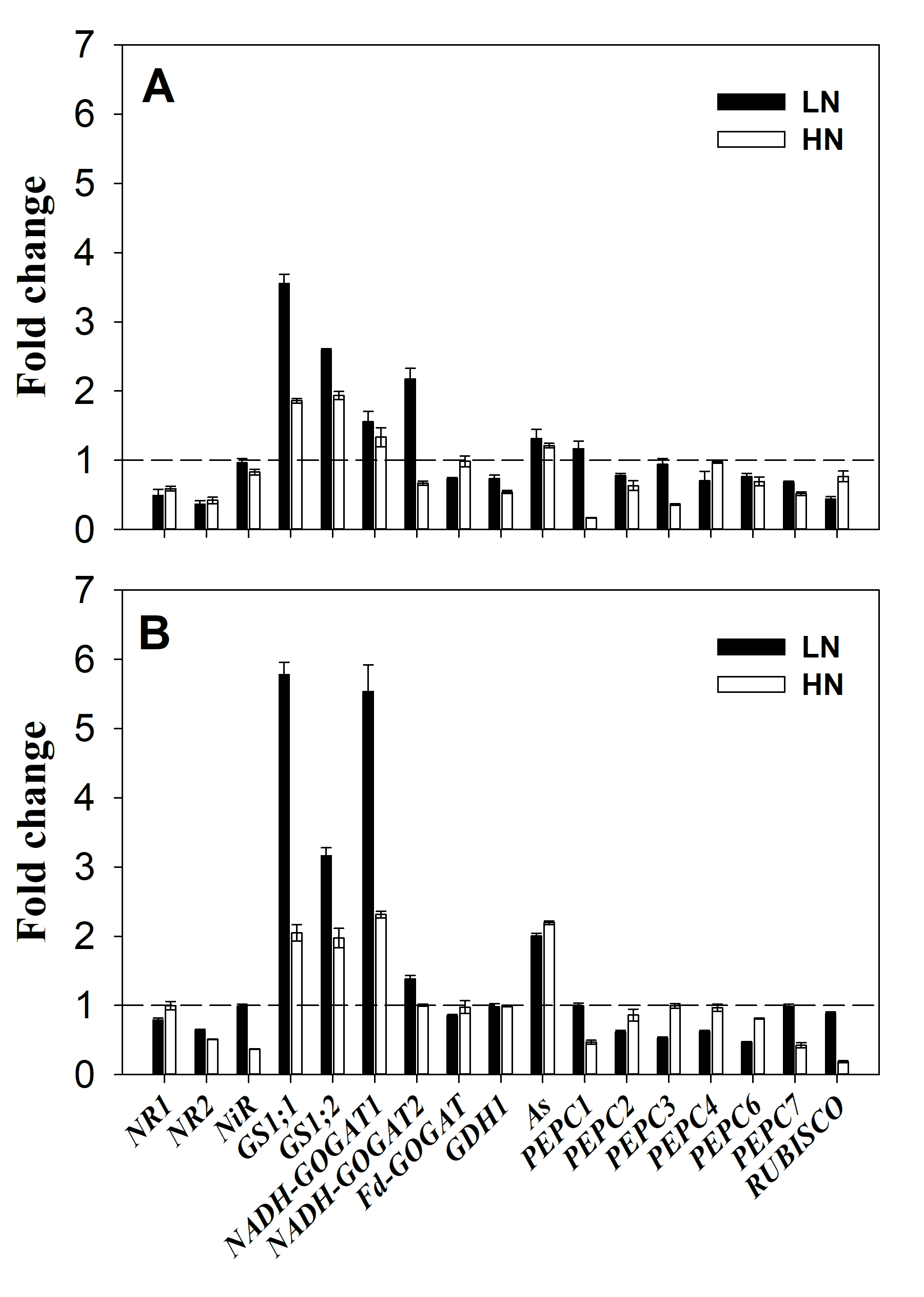

Supplement: S1 Fig — Fold change corresponding to the ratio of the gene expression level in transgenic lines TL35 (A) and TL44 (B) relative to the wildtype plants. (TIF) [file pone.0213504.s006.TIF]
